# Key Determinants of Human α-Defensin 5 and 6 for Enhancement of HIV Infectivity

**DOI:** 10.3390/v9090244

**Published:** 2017-08-29

**Authors:** Kimyata Valere, Wuyuan Lu, Theresa L. Chang

**Affiliations:** 1 Department of Microbiology and Molecular Genetics, Rutgers University, New Jersey Medical School, Newark, NJ 07103, USA; 06valere10@gmail.com; 2 Institute of Human Virology, University of Maryland School of Medicine, Baltimore, MD 21250, USA; wlu@ihv.umaryland.edu; 3 Public Health Research Institute, Rutgers University, New Jersey Medical School, 225 Warren Street, Newark, NJ 07103, USA

**Keywords:** defensins, HIV infectivity, attachment

## Abstract

Defensins are antimicrobial peptides important for mucosal innate immunity. They exhibit a broad spectrum of activity against bacteria, viruses, and fungi. Levels of α-defensins are elevated at the genital mucosa of individuals with sexually transmitted infections (STIs). Somewhat paradoxically, human α-defensin 5 and 6 (HD5 and HD6) promote human immunodeficiency virus (HIV) infectivity, and contribute to STI-mediated enhancement of HIV infection in vitro. Specific amino acid residues of HD5 and HD6 that are crucial for antimicrobial activities have been characterized previously; however, the key determinants of defensins responsible for enhancement of HIV infectivity are not known. Here, we have identified residues of HD5 and HD6 that are required for enhancement of HIV attachment and infection. Most of these residues are involved in hydrophobicity and self-association of defensins. Specifically, we found that mutant defensins L16A-HD5, E21me-HD5, L26A-HD5, Y27A-HD5, F2A-HD6, H27W-HD6, and F29A-HD6 significantly lost their ability to promote HIV attachment and infection. L29A mutation also reduced HIV infection-enhancing activity of HD5. Additionally, a number of mutations in charged residues variably affected the profile of HIV attachment and infectivity. One HD5 charged mutation, R28A, notably resulted in a 34–48% loss of enhanced HIV infectivity and attachment. These results indicate that defensin determinants that maintain high-ordered amphipathic structure are crucial for HIV enhancing activity. In a comparative analysis of the mutant defensins, we found that for some defensin mutants enhancement of HIV infectivity was associated with the reverse transcription step, suggesting a novel, HIV attachment-independent, mechanism of defensin-mediated HIV enhancement.

## 1. Introduction

Defensins are antimicrobial peptides important for innate mucosal immunity (reviewed in [1,2,3]). Individuals with sexually transmitted infections (STIs) have increased levels of defensins in genital secretions [4,5,6], suggesting a potential role of defensins in modulating human immunodeficiency virus (HIV) transmission. Human α-defensin 5 and 6 (HD5 and HD6) are constitutively expressed by Paneth cells in the small intestine [7], and their expression can be induced in cervicovaginal epithelial cells in response to STIs [8]. HD5 levels are increased in cervicovaginal lavage from women with bacterial vaginosis [4] and in urethral fluid from men infected with *Chlamydia trachomatis* or *Neisseria gonorrhoeae* [6] compared to individuals without STIs. Although HD5 and HD6 exhibit a broad spectrum of antimicrobial activities [9,10,11,12], we have previously shown that HD5 and HD6, paradoxically, promote HIV infectivity [8,13]. Knocking down HD5 or HD6 in vitro significantly reduces *Neisseria gonorrhoeae*-mediated enhancement of HIV infectivity [8]. These findings indicate that, in the setting of STIs, HIV may overcome the mucosal barrier by exploiting the increased levels of defensins produced by the host in response to bacterial pathogens.

HD5 and HD6 enhance HIV infectivity by aggregating virus particles, which promotes HIV attachment [13]. Linearized defensins in which cysteine residues are replaced by charge neutral isosteric α-aminobutyric acid do not promote HIV infectivity, indicating that defensin-mediated enhancement of HIV infectivity is structure-dependent [13,14]. Specific amino acid residues crucial for the antimicrobial activity of HD5 and HD6 have been identified [9,10,11,12]. Some residues in defensins appear to be critical for antimicrobial activity against multiple viral or bacterial pathogens, whereas, others are pathogen-specific. The molecular determinants of HD5 or HD6 associated with the HIV enhancing activity, however, remain uncertain. To address this issue, we have assembled a series of HD5 and HD6 mutants. Mutations that would affect charge, hydrophobicity, and self-association were selected (Table 1). We tested the effect of these mutations on three components of defensin-mediated HIV enhancing activity: HIV infection-enhancing activity, HIV attachment-enhancing activity, and late reverse transcription (RT) production. We found that mutations in residues involved in hydrophobicity and self-association significantly affected defensin-mediated HIV enhancing activity, whereas, mutations in charged residues produced variable effects on defensin-mediated HIV enhancing activity. We also found, unexpectedly, that some defensin mutants promoted HIV infectivity in an HIV attachment-independent manner.

## 2. Materials and Methods

### 2.1. Reagents

Materials for tissue culture including fetal bovine serum (FBS), Dulbecco’s Modified Eagle’s Medium (DMEM), and Dulbecco’s Phosphate Buffered Saline (PBS) were purchased from Sigma-Aldrich (Saint Louis, MO, USA). Lipofectamine 2000 Transfection Reagent was purchased from Invitrogen, Life Technologies (Norwalk, CT, USA).

HD5, HD6, and defensin mutants (Table 1) were chemically synthesized as previously described [15]. HD5 mutants generated using this synthesis procedure were Y4A, R9A, T12A, R13A, S15A, L16A, S17A, V19A, E21A, I22A, R25A, L26A, Y27A, R28A, L29Abu (aminobutyric acid), L29Nle (norleucine), and R32A [11]. Glutamic acid at position 21 of HD5 was replaced with *N*-methyl-glutamic acid for synthesis of E21me-HD5 [11]. L29A-HD5 was generated by chemical ligation with the HD5 pro-domain as described by Rajabi et al. [16]. HD6 mutants with single or double substitutions were generated as described previously [15]: H5N/H27N, H5A/H27A, H27A and H27W. Acetylation at the N-terminus of HD6 (Ac-HD6) or amidation at the C-terminus (HD6-HN2) were synthesized as described previously [17]. HD6 mutants with a single alanine substitution at residue 2 or 29 (i.e., F2A-HD6 and F29A-HD6) and wild type (WT) HD6 control were kindly provided by Elizabeth M. Nolan (Massachusetts Institute of Technology, Cambridge, MA, USA). These mutants were generated in pET28b-proHD6 by using Stratagene’s Quick-Change site directed mutagenesis (La Jolla, CA, USA), and expressed in *E. coli* TOP10 cells (Thermo Fisher Scientific, Waltham, MA, USA) to produce F2A-HD6, F29A-HD6, and WT HD6 [9]. The identity, purity, and disulfide bond connectivity of synthetic and bacteria-expressed defensins and mutants were confirmed as described previously [9,10,11,15,18].

### 2.2. HIV Infection

Replication-defective HIV-1_JR-FL_ Env-pseudotyped luciferase-expressing reporter viruses used in single-cycle infection assays were produced through Lipofectamine 2000 (Thermo Fisher Scientific) transfection of HEK293T cells with a plasmid encoding the envelope deficient HIV *NL4-3* virus and luciferase reporter gene (pNL-Luc-R+E-; gift of N. Landau, New York University, New York, NY, USA) along with a plasmid encoding HIV-1_JR-FL_ envelope as described previously [19,20]. After 24 h transfection, cells were cultured in serum-free media. The supernatant was collected 48 h after transfection, filtered through a sterilized syringe-driven filter (30 mm, polyethersulfone (PES) membrane, 0.22 μm), aliquoted and preserved at −80 °C.

For single-cycle infection assays, defensins were pre-incubated with serum-free HIV-1_JR-FL_ for 1 h at 37 °C. HeLa-CD4-CCR5 cells, provided by David Kabat (Oregon Health & Science University, Portland, OR, USA), were maintained in DMEM containing 10% FBS and seeded (2 × 10^4^ cells per well) in a white 96-well plate prior to infection with defensin-HIV-1_JR-FL_ mixture for 2 h at 37 °C. After infection, FBS at a final concentration of 10% was added to the wells, and cells were cultured for 2 days before lysis in passive lysis buffer (Promega, Madison, WI, USA). Luciferase activity (in relative light units (RLUs) was measured on a 2300 EnSpire Multimode Plate Reader (PerkinElmer, Waltham, MA, USA) or a MiniLumat LB9506 luminometer (EG&G Berthold, Oak Ridge, TN, USA). To summarize the results from multiple experiments, the average percent of infectivity compared to WT defensins was calculated using the formula: (RLU of defensin mutant-treated cells/RLU of WT defensin-treated cells) × 100.

### 2.3. HIV Attachment Assays

For HIV attachment assays, HeLa-CD4-CCR5 cells (3 × 10^4^ cells per well) were seeded in a 96-well plate and grown overnight. Defensins were pre-incubated with serum-free HIV-1_JR-FL_ for 1 h before HeLa-CD4-CCR5 cells were exposed to the defensin–virus mixture for 1 h at 4 °C. Unbound virus was washed off with cold PBS, and cells were lysed with 1% Triton X-100. AlphaLISA HIV-1 p24 kit (Perkin-Elmer) was used to measure cell-associated p24.

### 2.4. Late HIV Reverse Transcription Quantitative PCR

Total DNA was extracted from HIV-infected HeLa-CD4-CCR5 cells using the QIAamp DNA Blood Mini Kit (Qiagen, Germantown, MD, USA). The level of HIV late reverse transcription (RT) products was determined by quantitative real-time PCR analysis; each PCR reaction contained 100 ng total DNA, primers (200 nM each), and SYBR Green Master Mix (Qiagen). The primer sequences for HIV-1 late RT products were M667 R/gag forward (5′-GGCTAACTAGGGAACCCACTG-3′) and M661 R/gag reverse (5′-CCTGCCTCGAGAGAGCTCCACACTGAC-3′) [21]. The late RT standard curve consisted of 10-fold serial dilutions of pNL4-3.Luc.R-E ranging from 10 to 10^7^ copies. PCR cycling conditions included 95 °C for 10 min, then 40 cycles of 95 °C for 30 s, 55 °C for 30 s, and 72 °C for 30 s. Reactions were carried out and analyzed using Applied Biosystems StepOnePlus real-time PCR system (Agilent, Foster City, CA, USA). The detection limit of late RT DNA products was 10 copies.

### 2.5. Statistical Analysis

Statistical analysis was performed using a two-tailed Student’s *t*-test with Bonferroni correction or by one-way analysis of variance (ANOVA) with Bonferroni post-test; * *p* < 0.05 was considered significant. + *p* > 0.05 was considered insignificant after Bonferroni correction.

## 3. Results

### 3.1. Effect of HD5 Mutants on HIV Infectivity

**Figure 1:**
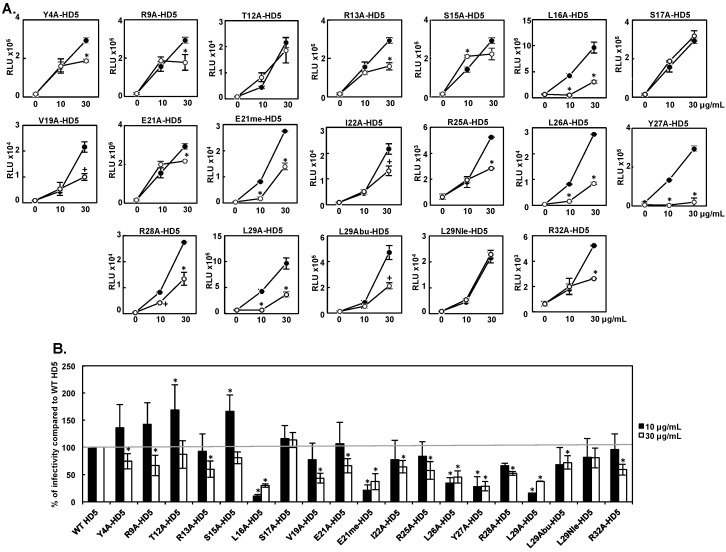
Effect of human α-defensin 5 (HD5) mutants on human immunodeficiency virus (HIV) infection-enhancing activity. Wild type (WT) HD5 or HD5 mutants were incubated with serum-free HIV-1_JR-FL_ for 1 h. HeLa-CD4-CCR5 cells were then incubated with the defensin–virus mixture for 2 h. After washing off unbound viruses, cells were cultured for 48 h before measuring infection by luciferase activity. (**A**) Results of a paired independent experiment for WT HD5 (filled circles) and each HD5 mutant (open circles) are shown. The relative light units (RLUs) of infected cells without defensin treatment were 410–860 or 2–7 × 10^4^ on a MiniLumat LB9506 luminometer (EG&G Berthold) or a PerkinElmer 2300 EnSpire Multimode Plate Reader, respectively. The signal of uninfected cells was not detectable. Data are means ± standard deviation (SD) of triplicate samples and represent seven independent experiments. * *p* < 0.05, WT HD5 versus HD5 mutants at the same concentration; Student’s *t*-test. After Bonferroni correction, the difference between WT HD5 versus V19A-HD5 (30 μg/mL), I22A-HD5 (30 μg/mL), R28-HD5 (10 μg/mL) and L29Abu-HD5 (30 μg/mL) was not significant (+ *p* > 0.05); (**B**) Data represent the average of the relative percent of enhanced infectivity by mutants to WT HD5 at the same concentration from seven independent experiments. * *p* < 0.05, WT HD5 versus HD5 mutants at the same concentration; one-way analysis of variance (ANOVA) with Bonferroni post-test.

To identify the molecular determinants critical for HD5-mediated enhancement of HIV-1 infectivity, we examined the effect of a panel of HD5 mutants on HIV-1 infection. WT HD5 or HD5 mutants at 10 or 30 μg/mL were incubated with serum-free HIV-1_JR-FL_ before adding the virus–defensin mixture to HeLa-CD4-CCR5 cells, and infection was assayed 48 h later. Results in Figure 1A showed a paired independent experiment for WT HD5 and each HD5 mutant, and a summary of seven independent experiments showing infection of mutants relative to WT HD5 is shown in Figure 1B. The E21me mutation, which disrupts defensin dimerization [11], significantly impaired HIV enhancing activity (79% at 10 μg/mL and 63% at 30 μg/mL); whereas, the E21A mutation only slightly reduced HD5 enhancing activity (34% reduction) and only at 30 μg/mL. Mutations of hydrophobic residues, L16A, L26A, Y27A, and L29A, reduced HD5-mediated enhancement of HIV infectivity from 54% to 90% compared to WT HD5. The L29 mutants, L29Abu-HD5 and L29Nle-HD5, which maintain hydrophobicity, had a slightly reduced or comparable HIV enhancing activity as WT HD5, whereas L29A-HD5, which abrogates hydrophobicity, lost enhancing activity. HIV enhancing activity of V19A-HD5 was reduced by 57% at 30 μg/mL, but S17A mutation had no significant effect on HIV enhancing activity at either concentration. T12A-HD5 and S15A-HD5 exhibited elevated HIV enhancing activity at 10 μg/mL, but had comparable levels of infectivity to WT HD5 at 30 μg/mL. Alteration of positively charged arginine residues also impaired HIV enhancing activity. HIV enhancing ability of other arginine mutants, R9A-HD5, R13A-HD5, R25A-HD5, R28A-HD5, and R32A-HD5, was reduced by 33% to 42% at 30 μg/mL, but was not significantly reduced at 10 μg/mL.

### 3.2. Effect of HD5 Mutants on HIV Attachment

**Figure 2:**
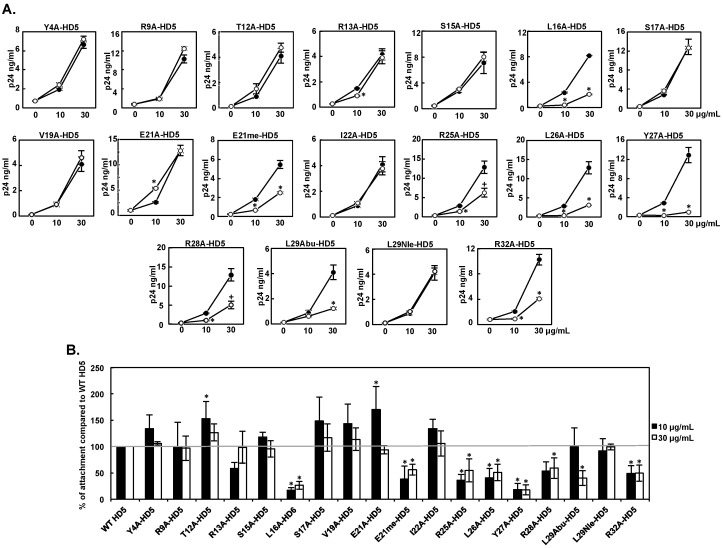
Effect of HD5 mutants on HIV attachment-enhancing activity. HD5 mutants were incubated with serum-free HIV-1_JR-FL_ for 1 h, and then the defensin–virus mixture was added to HeLa-CD4-CCR5 cells at 4 °C for 1 h. After washing off unbound viruses, cell-associated p24 was measured. (**A**) Results of a representative independent experiment for WT HD5 (filled circles) and each HD5 mutant (open circles) are shown. The baseline of cell-associated HIV p24 of control samples without defensin treatment was 130–779 ng/mL. Data are means ± SD of triplicate samples. * *p* < 0.05, WT HD5 versus HD5 mutants at the same concentration; 2-tailed Student’s *t*-test. After Bonferroni correction, the difference between WT HD5 versus R25A-HD5 and R28A-HD5 at 30 μg/mL was not significant (+ *p* > 0.05); (**B**) Data represent the average relative percent of each HD5 mutant on HIV attachment compared to WT HD5 at the same concentration from seven independent experiments. * *p* < 0.05, WT HD5 versus HD5 mutants at the same concentration; one-way ANOVA with Bonferroni post-test.

We have previously shown that HD5 enhances HIV infectivity through promoting HIV attachment [13]. To determine whether HD5 mutants had similar effects on HIV attachment, HIV-1_JR-FL_ was pre-treated with 10 or 30 μg/mL of HD5 mutants or WT HD5 for 1 h followed by incubation with HeLa-CD4-CCR5 cells at 4 °C for 1 h. Cells were washed, and HIV attachment was assessed by determining the levels of cell-associated HIV p24. Results in Figure 2A showed a paired independent experiment for WT HD5 and each HD5 mutant, and a summary of seven experiments is shown in Figure 2B. Similar to the profile of HIV infectivity, L16A, E21me, L26A, and Y27A mutations had a deleterious effect on enhancing HIV attachment at both concentrations (44% to 83% reduction), whereas Y4A, S17A, and L29Nle mutations had no significant effect at both concentrations. The ability of R28A-HD5 to promote HIV attachment was moderately reduced (41% at 30 μg/mL) similar to its HIV enhancing effect. T12A-HD5 at 10 μg/mL had an enhanced ability to promote HIV attachment (53% increase) and infectivity (69% increase). However, not all mutants exhibited consistent profiles of HIV infectivity and attachment (Table 2). For example, R9A-HD5, R13A-HD5, V19A-HD5, E21A-HD5, and I22A-HD5 exhibited reduced HIV infection-enhancing activity at 30 μg/mL, but did not differ from WT HD5 with respect to HIV attachment-enhancing activity at the same concentration. This suggests that HIV infection-enhancing activity is not simply a consequence of increased HIV attachment. Conversely, R25A-HD5 and R32A-HD5 had impaired HIV attachment-enhancing activity (45% to 64% reduction at 10 and 30 μg/mL), but exhibited impaired HIV infection-enhancing activity only at 30 μg/mL. The fact that 10 μg/mL of R25A and R32A mutations exhibited reduced HIV attachment-enhancing activity, but not reduced HIV infection-enhancing activity, suggests that HD5-mediated HIV infection-enhancing activity occurs, at least in part, through an HIV attachment-independent mechanism.

### 3.3. Some HD5 Mutants Affect HIV Reverse Transcription

**Figure 3:**
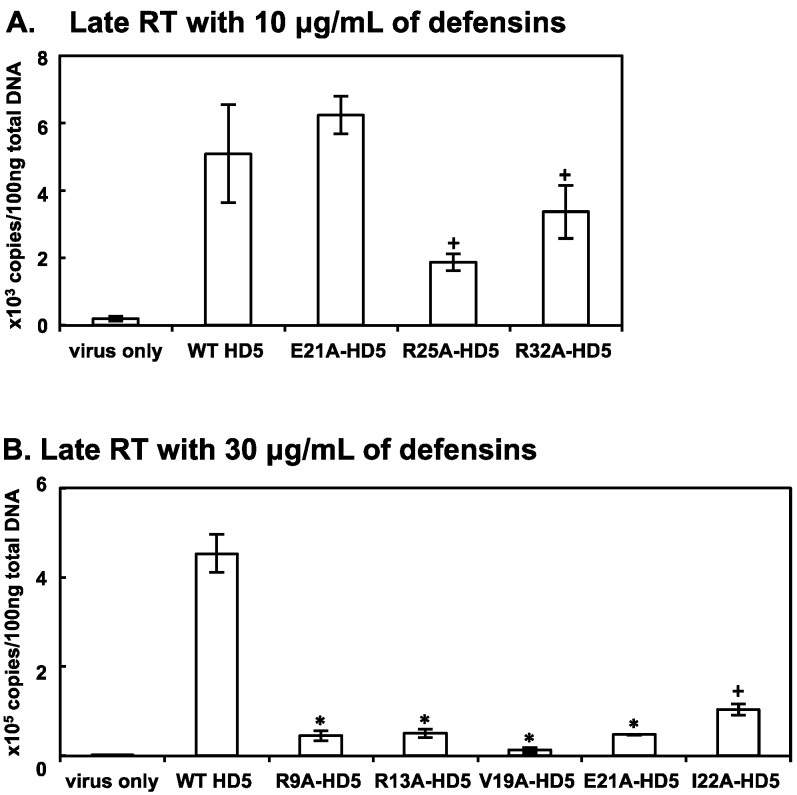
Effect of selected HD5 mutants on HIV reverse transcription. WT HD5 or HD5 mutants at 10 μg/mL (**A**) or 30 μg/mL (**B**) were pre-incubated with serum-free HIV-1_JR-FL_ for 1 h followed by infection of HeLa-CD4-CCR5 cells. After washing off unbound viruses, cells were cultured for an additional 12 h at 37 °C before preparation of total DNA for the analysis of HIV-1 late RT by quantitative real-time PCR. The baseline of RT copy numbers from cells infected by HIV without defensins was 204–236. * *p* < 0.05, WT HD5 versus HD5 mutants at the same concentration; Student’s *t*-test. The difference between WT HD5 versus R25A-HD5 (10 μg/mL), R32A-HD5 (10 μg/mL), and I22A-HD5 (30 μg/mL) was not significant after Bonferroni correction, (+ *p* > 0.05).

We speculated that HD5 mutants that exhibited inconsistent profiles for HIV attachment and infection may affect post-attachment steps in the HIV life cycle. Thus, we examined effects on HIV reverse transcription of R9A-HD5, R13A-HD5, V19A-HD5, E21A-HD5, I22A-HD5, R25A-HD5, and R32A-HD5 at the same concentrations that produced the inconsistent attachment and infection profiles. As in previous experiments, HIV-1_JR-FL_ was pre-incubated with WT HD5 or HD5 mutants before being added to cells. After washing off unbound virus, cells were incubated for 12 h before extraction of total DNA. The levels of late RT products were determined by quantitative PCR analysis (Figure 3). We found that HD5 mutants that exhibited impaired HIV attachment-enhancing activity at 10 μg/mL, but retained HIV infection-enhancing activity infectivity (i.e., R25A-HD5 and R32A-HD5), also exhibited reduced late RT production compared to WT HD5 although the difference was not significant after Bonferroni correction. Other HD5 mutants, notably E21A-HD5 at 10 μg/mL, exhibited increased HIV attachment-enhancing activity, but retained levels of RT products and HIV infectivity that were comparable to WT HD5. Some HD5 mutants, when used at 30 μg/mL, exhibited similar levels of HIV attachment-enhancing activity as WT HD5, but significantly impaired HIV infection-enhancing activity and late RT production (i.e., R9A-HD5, R13A-HD5, V19A-HD5, E21A-HD5, and I22A-HD5). In aggregate, the data shown in Figure 3 suggest that HD5 defensin-mediated HIV enhancing activity includes attachment-dependent and attachment-independent mechanisms. The latter appear to include effects on reverse transcription.

### 3.4. Effect of HD6 Mutants on HIV Infection-Enhancing Activity

**Figure 4:**
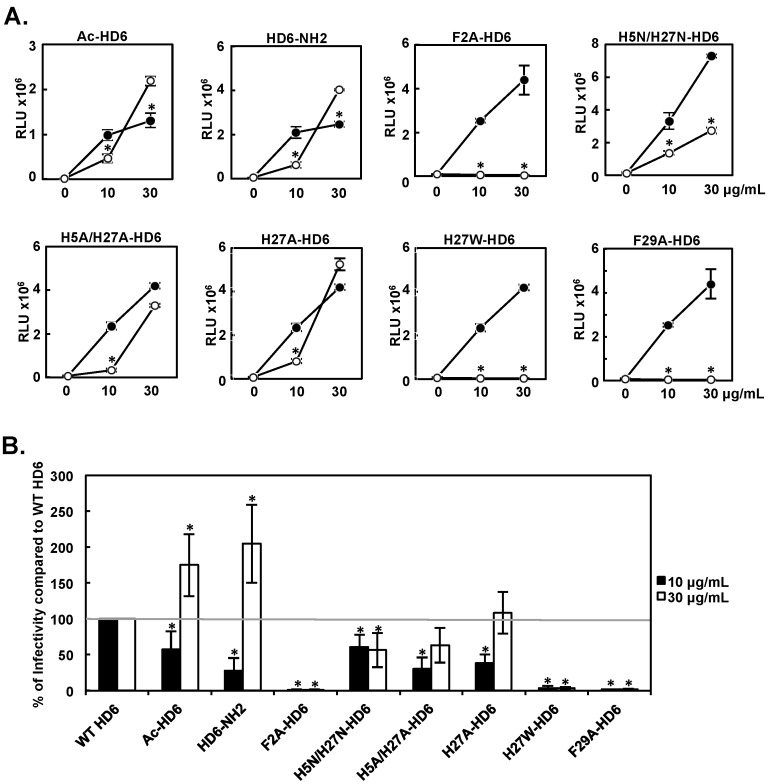
Effect of HD6 mutants on HIV infection-enhancing activity. WT HD6 or HD6 mutants were incubated with serum-free HIV-1_JR-FL_ for 1 h followed by infection of HeLa-CD4-CCR5 cells. Luciferase activity was measured 48 h after infection. (**A**) Results of a representative independent experiment for each HD6 mutant (open circles) and WT HD6 (filled circles) are shown. The RLUs of cells infected by HIV without defensins were 13,332–76,853. Data are means ± SD of triplicate samples. * *p* < 0.05, WT HD6 versus HD6 mutants at the same concentration by Student’s *t*-test. (**B**) Data represent the average relative percent of enhanced HIV infectivity by mutants to WT HD6 at the same concentrations from more than three sets of experiments for each mutant. * *p* < 0.05, WT HD6 versus HD6 mutants at the same concentration by one-way ANOVA with Bonferroni post-test.

HD5 and HD6 promote HIV infectivity via distinct mechanisms [13]. To determine residues in HD6 important for HIV enhancement, we performed similar experiments with HD6 mutants as we did with HD5 mutants. WT HD6 or HD6 mutants at 10 or 30 μg/mL were pre-incubated with serum-free HIV-1_JR-FL_ for one hour before addition to cells. HIV infection was determined 48 h later by a luciferase-based single-cycle infection assay. Results in Figure 4A showed a paired independent experiment for WT HD6 and each HD6 mutant, and a summary of results from more than three sets of independent experiments for each mutant are summarized in Figure 4B. At 30 μg/mL, Ac-HD6 and HD6-NH2 mutants exhibited elevated HIV infection-enhancing activity, but at 10 μg/mL there was a negative effect. Among the other HD6 mutants tested, F2A, H5N/H27N, H27W, and F29A exhibited reduced HIV infection-enhancing activity at both concentrations tested (35% to 99% reduction). However, H27A-HD6 and H5A/H27A-HD6 exhibited reduced HIV enhancing activity only at 10 μg/mL (61% and 70% reduction, respectively), but had no significant effect when used at 30 μg/mL.

### 3.5. Effect of HD6 Mutants on HIV Attachment-Enhancing Activity

**Figure 5:**
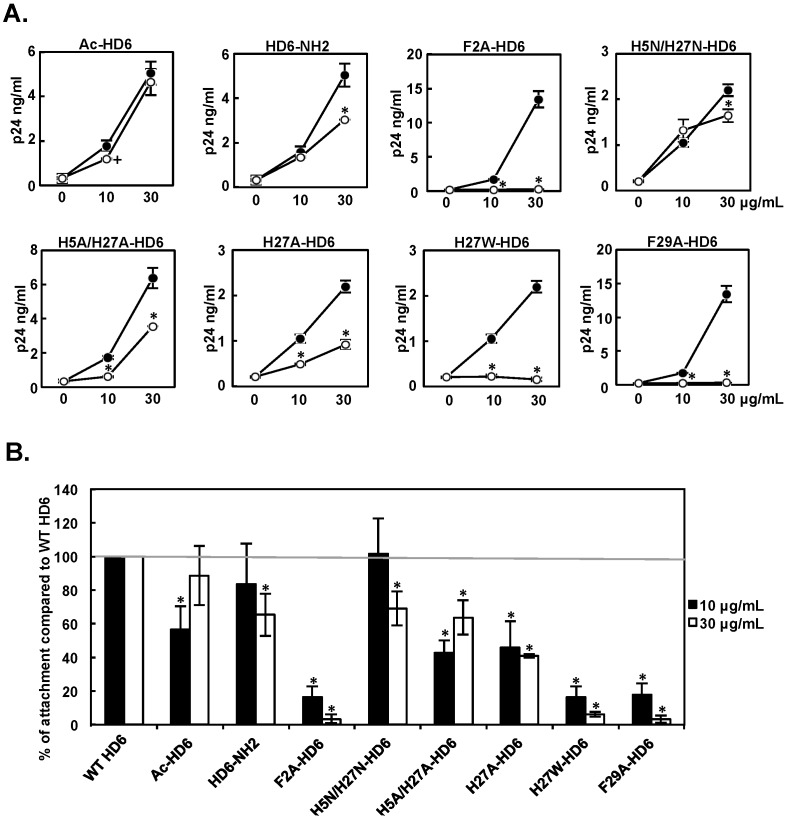
Effect of HD6 mutants on HIV attachment-enhancing activity. WT HD6 or HD6 mutants were pre-incubated with serum-free HIV-1_JR-FL_ before incubation with HeLa-CD4-CCR5 cells at 4 °C for 1 h. After washing off unbound viruses, cell-associated p24 was measured. (**A**) Results of a representative independent experiment for WT HD6 (filled circles) and each HD6 mutant (open circles) are shown. The baseline HIV p24 value (HIV without defensin) was 204–305. Data are means ± SD of triplicate samples and represent six independent experiments. * *p* < 0.05, WT HD6 versus HD6 mutants at the same concentration; 2-tailed Student’s *t*-test. After Bonferroni correction, the difference between WT HD6 and Ac-HD6 at 10 μg/mL was not significant (+ *p* > 0.05). (**B**) Data represent the average relative percent of enhanced HIV attachment by mutants compared to WT HD6 from six independent experiments. * *p* < 0.05, WT HD6 versus HD6 mutants at the same concentration by one-way ANOVA with Bonferroni post-test.

We then determined the effect of HD6 mutants on HIV attachment as described in Figure 2. Results in Figure 5A showed individual representative experiments, and results from six independent experiments are summarized in Figure 5B. F2A, H27W, and F29A mutants significantly lost their ability to promote HIV attachment at both concentrations tested. The extent of the reduction in HIV attachment-enhancing activity ranged from 83% to 97% compared to WT HD6. H5A/H27A and H27A mutants also exhibited reduced HIV attachment-enhancing activity at both concentrations (32–58% reduction). H5N/H27N mutant had reduced HIV attachment-enhancing activity at 30 μg/mL, but not at 10 μg/mL. HD6-NH2 exhibited reduced HIV attachment-enhancing activity at 30 μg/mL (by 35%), but its HIV infection-enhancing activity was further increased (Table 3). Although Ac-HD6 and HD6-NH2 were the only two mutants that exhibited increased HIV infection-enhancing activity (Figure 4B), neither of these exhibited any increase in HIV attachment-enhancing activity. H27A-HD6 exhibited reduced HIV infection-enhancing activity at 10 μg/mL, but not at 30 μg/mL. However, it exhibited reduced HIV attachment-enhancing activity at both concentrations (54% to 59% reduction). In aggregate, these data suggest that some HD6 mutants promote HIV infection through attachment-independent pathways.

### 3.6. Effect of HD6 Mutations on Reverse Transcription

**Figure 6:**
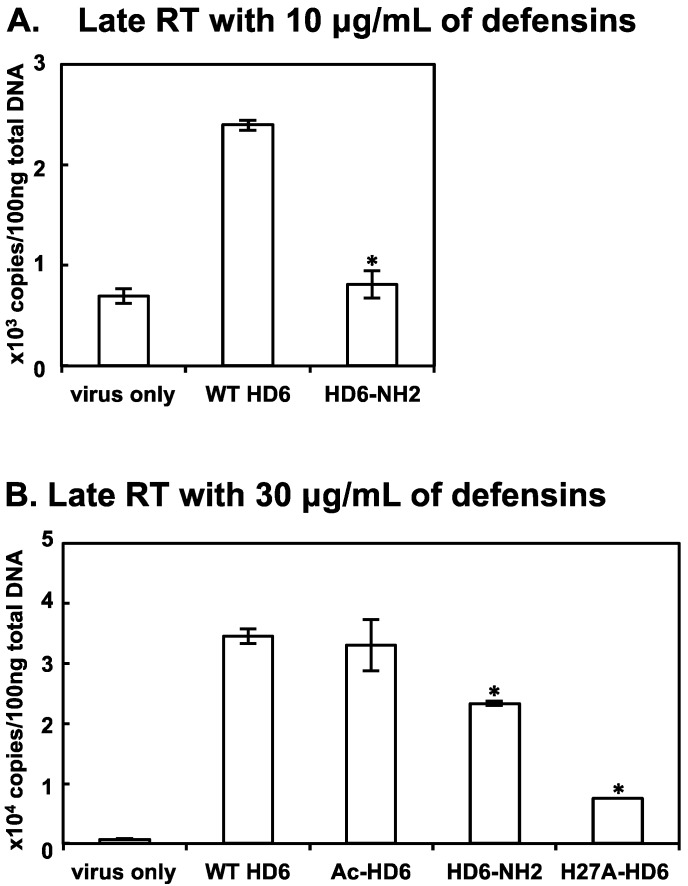
Effects of selected HD6 mutants on HIV reverse transcription. WT HD6 or HD6 mutants were pre-incubated with serum-free HIV-1_JR-FL_ for 1 h followed by infection of HeLa-CD4-CCR5 cells at 37 °C for 2 h. After washing off unbound viruses, cells were cultured for 12 h before preparation of total DNA. The levels of HIV-1 late RT products were determined by quantitative real-time PCR. The baseline of RT copy numbers from cells infected by HIV without defensins was 679–700. * *p* < 0.05, WT HD6 versus HD6 mutants at the same concentration; Student’s *t*-test.

To ascertain whether HD6 mutants that exhibited incongruent attachment-enhancing and infection-enhancing profiles affected other steps in the HIV life cycle, we determined the effects of these mutants on HIV reverse transcription (Figure 6A,B). Among these, Ac-HD6 at 30 μg/mL exhibited comparable HIV attachment-enhancing activity (Figure 5A,B) and late RT production (Figure 6B) as WT HD6, but Ac-HD6 HIV infection-enhancing activity (Figure 4A,B) was greater than WT HD6, indicating that this HD6 mutant may promote HIV infection by acting on a step after reverse transcription. Conversely, at 10 μg/mL, HD6-NH2 did not affect HIV attachment-enhancing activity (Figure 5A,B), but did exhibit reduced HIV infection-enhancing activity (Figure 4A,B) and reduced late RT production (Figure 6A), suggesting that reduced HIV infection-enhancing activity by HD6-NH2 involved its inhibitory effect on reverse transcription. Although at 30 μg/mL, H27A-HD6 and HD6-NH2 exhibited comparable or greater levels of HIV infection-enhancing activity as WT HD6, they exhibited reduced HIV attachment-enhancing activity and reduced late RT production (Figure 6B), suggesting that these mutants may also act on a step after reverse transcription that increases their HIV infection-enhancing activity.

## 4. Discussion

In this study, we identified key determinants of HD5 and HD6 that affect their HIV infection-enhancing and attachment-enhancing activities. Analysis of defensin mutants that have alterations of charge, hydrophobicity, or dimerization indicated that L16A, E21me, L26A, and Y27A mutations in HD5 impaired defensin-mediated enhancement of HIV attachment and infection. F2A, H27W, and F29A mutations in HD6 were detrimental to HD6-mediated enhancement of HIV attachment and infection. Although we previously showed that WT HD5 and HD6 enhance HIV infectivity by promoting HIV attachment [13], here, we also identified defensin mutants that had different profiles of HIV attachment-enhancing and infection-enhancing activities, indicating that these defensin mutants promoted HIV infectivity through an attachment-independent mechanism. Indeed, we found that some defensin mutants affected the HIV life cycle at the step after HIV attachment including reverse transcription.

The hydrophobicity and charge of HD5 have been shown to be critical for its antimicrobial activity [11,12]. We found that not all mutations in hydrophobic or charged residues had the same impact on the defensin activity for HIV infection. For example, the activity of Y4A-HD5 was similar to WT HD5. At some tested concentrations, R9A-HD5, R13-HD5, and R32A-HD5 mutations did not have a significant impact on HIV attachment-enhancing and infection-enhancing activities. However, among mutants that showed a decrease in HIV enhancing activity, mutations affecting hydrophobicity generally had a greater impact (50 to 90 percent reduction) than mutations affecting charge (33 to 42 percent reduction). In this regard, a L29A-HD5 mutation, which reduced hydrophobicity, resulted in impaired HIV attachment-enhancing and infection-enhancing activity; whereas, L29Abu-HD5 and L29Nle-HD5 mutations, which retained hydrophobicity [11], had a less negative impact on HIV enhancing activity.

The critical determinants of HD5 that mediate antiviral activity against adenovirus and HPV [12,22] are distinct from those that mediate HIV attachment-enhancing and infection-enhancing activities. For example, R28A-HD5 is the most deleterious arginine mutant with respect to anti-adenovirus activity of HD5 [22]; however, this mutant only lost about 50 percent of its HIV attachment-enhancing and infection-enhancing activities. While all R to A mutants (R9A, R13A, R25A, R28A, and R32A) affected HIV infection-enhancing activities to some extent when applied at a higher concentration, when used at a lower concentration, HIV infection-enhancing activities of R9A, R13A, R25A, and R32A were similar to WT HD5. Lehrer and colleagues have shown that R to A mutants (R9,28A-HD5 and R13,32A-HD5) have reduced affinity for HIV gp120 and a weaker ability to self-assemble than WT HD5 [23]. However, reduced binding ability of mutants to HIV gp120 does not easily explain the HIV enhancing activities of these mutants at different concentrations in our studies. One possible explanation might be that, at the different concentrations employed, defensins in a monomeric or multimeric form may interact with HIV particles differently, and it is this difference in interaction which leads to differential profiles of HIV enhancing activity.

Although most HD5 mutants affected the activity of HD5 in a pathogen-dependent matter, mutations of tyrosine (Y) residues appeared to have a common effect on HD5 function. For example, the antiviral activity of Y27A-HD5 against adenovirus and HPV is weaker than that of Y4A-HD5 [12]. Similarly, we found that the reduction in HIV enhancing activity of Y27A-HD5 was greater than that of Y4A-HD5, suggesting that the Y27A mutation may have an impact on HD5 structure that affects both its antimicrobial function and its HIV enhancing function. The Y27 residue is located near the C-terminus of HD5, and is critical to maintaining the functionality and orientation of surface-exposed residues [12]. The C-terminal hydrophobic and positively charged residues of HD5 are also important for its bactericidal activity [11].

We have previously shown that linearized HD5 and HD6, even in the absence of any change in charge, have no HIV enhancing activity [13]. The cationic nature of defensins contributes to their antimicrobial function (reviewed in [24]), but our results indicate that charge-changing mutations often resulted in a moderate decrease in HIV infection-enhancing activity. Thus, charged residues presented in a proper structure, rather than the charge alone, are important for the optimal function of defensins. We also found that the loss of a negatively charged residue in the E21A mutant, which should lead to a more cationic overall charge, had an adverse effect on the HIV enhancing activity of HD5 at 30 μg/mL, but not at 10 μg/mL, suggesting that the cationic nature of defensins is less important to their HIV enhancing activity than to their antimicrobial functions.

Dimerization or oligomerization of defensins play an important role in their functions [3,11,12,22,25]. For example, an E21me mutation disrupts dimerization of HD5 and negatively impacts the antibacterial and antiviral activities [12,22]. Similarly, the E21me mutation significantly reduced the HIV infection-enhancing activity of HD5, indicating an important role of HD5 dimerization in defensin function. Disruption of HD6 oligomerization by an H27W mutation, impairs HD6 antibacterial activity [9,10]. Mutations of hydrophobic residues F2A and F29A are also detrimental to HD6 oligomerization and impaired antibacterial activity [9]. Our results showed that H27W, F2A, and F29A mutations significantly weakened the HIV attachment-enhancing and infection-enhancing activity of HD6. The H27W mutation was more detrimental than H5N/H27N, H5A/H27A, or H27A mutations. It is possible that H27A-HD6 retains its ability to self-associate and to form elongated fibril structures, whereas H27W-HD6 forms fewer and smaller fibrils [9].

Analysis of specific defensin mutants that exhibited distinct HIV infection-enhancing activity and HIV attachment-enhancing activity profiles allowed us to identify defensin variants that enhanced HIV infectivity through an HIV attachment-independent mechanism (Table 2 and Table 3). We found that mutants R9A-HD5, R13A-HD5, V19A-HD5, E21A-HD5, and I22A-HD5 had reduced late RT production and reduced HIV infection-enhancing activity, but these mutants exhibited HIV attachment-enhancing activity comparable to WT HD5. Thus, it is possible that these mutants may act on the step of reverse transcription or a step before reverse transcription (i.e., fusion) to modulate HIV infectivity. We also identified mutations that acted on steps after reverse transcription. For example, the HIV attachment-enhancing activity and late reverse transcription of H27A-HD6 at 30 μg/mL was lower than WT HD6, but had comparable HIV infection-enhancing activity to WT HD6. Similarly, HIV attachment-enhancing activity and reverse transcription of HD6-NH2 at 30 μg/mL were reduced compared to WT HD6, but its infection-enhancing activity was two-fold higher than WT HD6. The HIV infection-enhancing activity of Ac-HD6 at 30 μg/mL was higher than WT HD6, but HIV attachment-enhancing activity and reverse transcription were comparable. Although we have previously shown that pretreatment of HeLa-CD4-CCR5 cells with WT HD5 or HD6 has a minimal effect on HIV infection [13], defensin mutants may function intracellularly to affect steps post-HIV attachment. Human β-defensin 2 (HBD2) and HBD3 have been shown to co-internalize with HIV and to reduce viral infectivity intracellularly [26]. HD5, but not HD6, exhibits post-entry anti-HSV2 activity by interfering with gene expression and DNA replication [27]. It remains to be determined whether HD5 or HD6 mutants co-internalize with HIV to modulate viral infection in infected cells and whether the defensin–virus complex may trigger cell signaling pathways to promote HIV infection.

In summary, our results identified key determinants of defensins for HIV attachment-enhancing and infection-enhancing activities. Mutations altering hydrophobicity and self-association significantly impaired defensin-mediated HIV attachment-enhancing and infection-enhancing activities. Additionally, we identified a novel mechanism by which HD5 and HD6 mutants modulated HIV infectivity after the step of viral attachment. Together with previous reports, we have shown that determinants involved in maintaining structure are important for overall function of defensins, but some residues are involved in pathogen-specific functions.

## Figures and Tables

**Table 1:** Human α-defensin 5 and 6 (HD5 and HD6) mutants and properties of mutated amino acid residues. NA: information is not available.

Defensin Mutants	Property
Y4A-HD5	hydrophobicity
R9A-HD5	cationicity
T12A-HD5	NA
R13A-HD5	cationicity
S15A-HD5	NA
L16A-HD5	hydrophobicity
S17A-HD5	NA
V19A-HD5	hydrophobicity
E21A-HD5	anionicity
E21me-HD5	dimerization
I22A-HD5	hydrophobicity
R25A-HD5	cationicity
L26A-HD5	hydrophobicity
Y27A-HD5	hydrophobicity
R28A-HD5	cationicity
L29A-HD5	hydrophobicity
L29Abu-HD5	hydrophobicity
L29Nle-HD5	hydrophobicity
R32A-HD5	cationicity
Ac-HD6	NA
HD6-NH2	NA
F2A-HD6	self-association/hydrophobicity
H5N/H27N-HD6	polarity
H5A/H27A-HD6	polarity
H27A-HD6	self-association
H27W-HD6	self-association
F29A-HD6	self-association/hydrophobicity

**Table 2:** Summary of the effect of HD5 mutants on HIV attachment-enhancing activity and infection-enhancing activity. Data are means ± SD of seven independent experiments, and represent the average relative percent of HIV attachment or infectivity by HD5 mutants compared to WT HD5 at the same concentration.

	Attachment 10 μg/mL	Infectivity 10 μg/mL	Attachment 30 μg/mL	Infectivity 30μg/mL
**WT HD5**	100 ± 0	100 ± 0	100 ± 0	100 ± 0
**Y4A-HD5**	134 ± 26	136 ± 42	106 ± 3	75 ± 14
**R9A-HD5**	100 ± 46	142 ± 40	97 ± 23	67 ± 19
**T12A-HD5**	153 ± 32	169 ± 47	127 ± 16	87 ± 25
**R13A-HD5**	59 ± 11	92 ± 32	98 ± 30	60 ± 15
**S15A-HD5**	118± 9	166 ± 30	95 ± 16	81 ± 11
**L16A-HD5**	17 ± 5	10 ± 3	27 ± 7	30 ± 3
**S17A-HD5**	148 ± 46	116 ± 24	117 ± 26	113 ± 14
**V19A-HD5**	144 ± 37	77 ± 31	114 ± 21	43 ± 9
**E21A-HD5**	171 ± 44	107 ± 40	94 ± 8	66 ± 13
**E21me-HD5**	38 ± 25	21 ± 10	56 ± 10	37 ± 15
**I22A-HD5**	134 ± 18	77 ± 36	106 ± 24	64 ± 11
**R25A-HD5**	36 ± 11	83 ± 27	55 ± 22	58 ± 16
**L26A-HD5**	41 ± 18	35 ± 10	51 ± 16	46 ± 11
**Y27A-HD5**	19 ± 11	28 ± 18	18 ± 9	29 ± 9
**R28A-HD5**	54 ± 17	66 ± 5	59 ± 19	52 ± 3
**L29A-HD5**	NA	16 ± 0	NA	38 ± 0
**L29Abu-HD5**	99 ± 36	68 ± 32	40 ± 14	72 ± 12
**L29Nle-HD5**	92 ± 23	82 ± 35	99 ± 6	81 ± 18
**R32A-HD5**	49 ± 15	96 ± 29	50 ± 15	59 ± 10

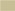
: Comparable activity for HIV attachment and infectivity at both concentrations to WT HD5; 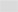: Comparable activity for attachment or infectivity at a specific concentration to WT HD5; 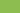: Reduced activity for HIV attachment and infectivity at a specific concentration compared to WT HD5; 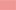: Reduced activity, but not consistent profiles of attachment and infectivity, compared to WT HD5 (*p* < 0.05); 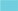: Enhanced activity when compared to WT HD5 (*p* < 0.05).

**Table 3:** Summary of the effect of HD6 mutants on HIV attachment-enhancing activity and infection-enhancing activity. Data are means ± SD of six independent experiments, and represent the average relative percent of HIV attachment or infectivity by HD6 mutants compared to WT HD6 at the same concentration.

	Attachment 10 μg/mL	Infectivity 10 μg/mL	Attachment 30 μg/mL	Infectivity 30 μg/mL
**WT HD6**	100 ± 0	100 ± 0	100 ± 0	100 ± 0
**Ac-HD6**	57 ± 14	58 ± 24	87 ± 19	175 ± 43
**HD6-NH2**	99 ± 14	28 ± 17	65 ± 12	205 ± 55
**F2A-HD6**	17 ± 6	1 ± 1	4 ± 3	1 ± 0
**H5N/H27N-HD6**	102 ± 21	65 ± 16	69 ± 9	60 ± 24
**H5A/H27A-HD6**	43 ± 7	30 ± 16	68 ± 10	63 ± 24
**H27A-HD6**	46 ± 16	39 ± 12	41 ± 1	108 ± 29
**H27W-HD6**	17 ± 6	4 ± 3	6 ± 1	3 ± 2
**F29A-HD6**	18 ± 7	2 ± 0	3 ± 2	2 ± 1

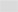
: Comparable activity for attachment or infectivity at a specific concentration to WT HD6; 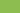: Reduced activity for HIV attachment and infectivity at a specific concentration compared to WT HD6; 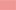: Reduced activity, but not consistent profiles of attachment and infectivity, compared to WT HD6 (*p* < 0.05); 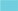: Enhanced activity when compared to WT HD6 (*p* < 0.05).
